# Immunologic Control of Disseminated Aichi Virus Infection in X-Linked Agammaglobulinemia by Transplantation of TcRαβ-Depleted Haploidentical Hematopoietic Cells

**DOI:** 10.1007/s10875-022-01314-5

**Published:** 2022-07-05

**Authors:** Zivile Bekassy, Mats Ehinger, Linda Nyberg Pronk, Cornelis Jan Pronk, for the SMPOT group, Zivile Bekassy, Mats Ehinger, Linda Nyberg Pronk, Cornelis Jan Pronk, Dominik Turkiewicz, Martin Lindström, Ladislav Król, Nicholas Brodszki

**Affiliations:** 1 Department of Pediatrics, Clinical Sciences Lund, Lund University, Lund, Sweden; 2 Department of Pediatric Nephrology, Skåne University Hospital, Lasarettsgatan 48, 221 85 Lund, Sweden; 3 Department of Clinical Sciences, Division of Pathology, Lund University, Lund, Sweden; 4 Capio City Clinic, Kristianstad, Sweden; 5 Chilhood Cancer Center, Skåne University Hospital, Lund, Sweden; 6 Wallenberg Centre Molecular Medicine and Division Molecular Hematology, Lund University, Lund, Sweden

To the Editor,

We report a rare case of a chronic disseminated Aichi virus infection in a pediatric patient with X-linked agammaglobulinemia (XLA) that was controlled upon HLA-haploidentical hematopoietic cell transplantation (haplo-HCT).

XLA is a rare primary immune deficiency caused by defects in the gene encoding Bruton’s tyrosine kinase (BTK) and characterized by the absence of circulating B-cells and absent or very low serum immunoglobulin (Ig) levels. Our patient was diagnosed at the age of 1.5 years, based on absent B-lymphocytes (CD19^+^  < 0.01 × 10^9^), undetectable levels of IgG (< 0.39 g/L), and markedly reduced levels of IgM (0.2 g/L) and IgA (0.1 g/L), and was genetically verified by detection of a mutation in the *BTK* gene (c.1816C > T, p.Arg562Trp). Ig replacement therapy was commenced with subcutaneous Ig infusions maintaining mean IgG trough levels at 7 g/L. The patient had no hepatosplenomegaly, growth and development were uneventful, apart from recurrent purulent conjunctivitis, intermittent asthma, and mild eczema.

At the age of 13.5 years, his condition deteriorated, with recurrent episodes of fever and cough, weight loss, and unilateral bronchiectasis. Increasing creatinine levels (120–160 µmol/L, normal range 37–72) and decreasing glomerular filtration rate (GFR) as measured by iohexol clearance of 37–40 mL/min/1.7 m^2^ (86–124) were observed at age 14 years. The mean IgG trough level was low at 4.4 g/L despite IgG replacement of 140 mg/kg bodyweight/week. Ultrasound showed hyperechoic enlarged kidneys (> 99%-percentile). Repeated kidney biopsies (numbers N#1&2) revealed massive tubulointerstitial nephritis with predominant CD8^+^, CD52^+^ T-cell infiltration. Immunohistochemical analysis of this lymphoid infiltration did not show any aberrant phenotype. However, PCR analysis for TCR-rearrangement showed a monoclonal origin of these kidney-residing lymphocytes (Fig. 1g, upper panel). This clone was also detected in the peripheral blood (Fig. 1g, lower panel) and in the bone marrow samples. Extensive diagnostic workup could not identify any pathogen as a possible triggering factor (Supplementary Table). Tubulointerstitial nephritis caused by intravenous Ig therapy has been described in an adult XLA patient [1], but no IgG deposition was found in the kidneys of our patient. Increased metabolism in the kidneys, thymus, and bone marrow could be visualized by positron emission tomography imaging. However, repeated morphologic and immunophenotypic analyses of the T-cell infiltrate in the kidney (N#3) (Fig. 1a–b) and bone marrow were not typical for peripheral T-cell lymphoma, hence the criteria for lymphoma were never fulfilled. The boy was considered to have a “monoclonal T-cell expansion in the kidney, bone marrow and blood of uncertain malignant potential.”

**Fig. 1:**
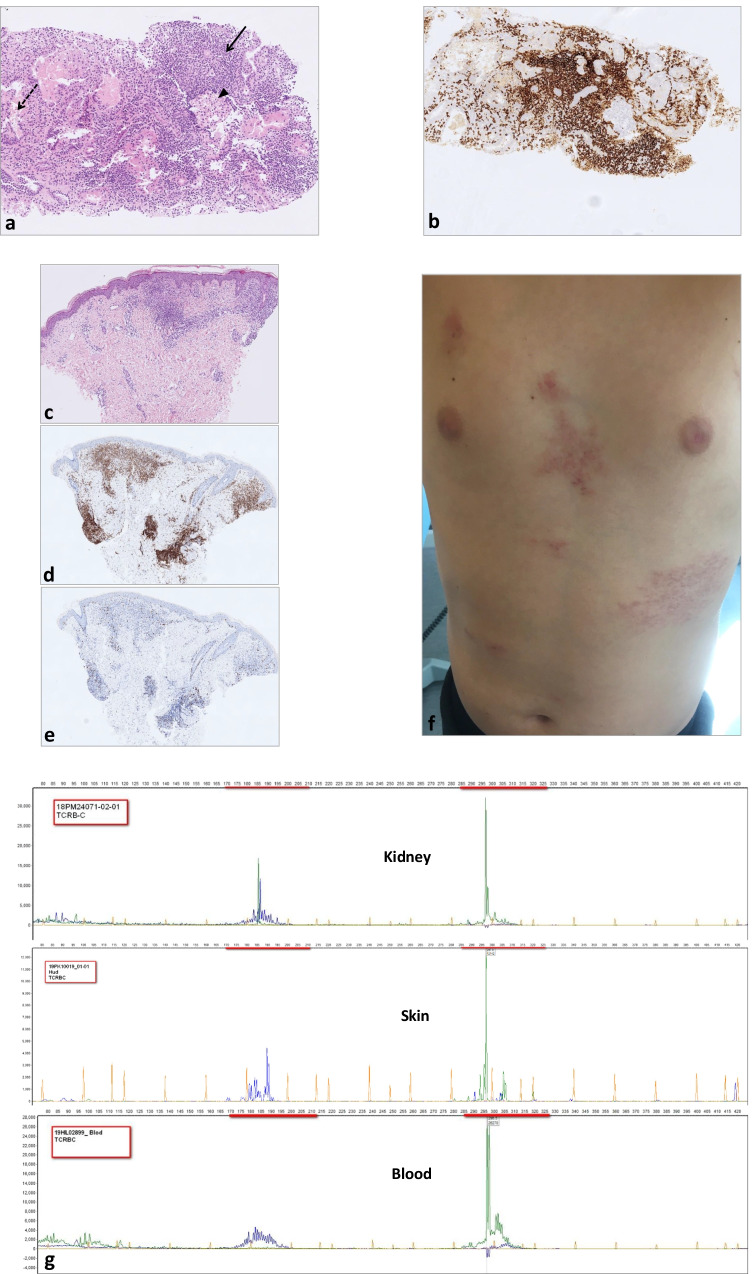
Diagnostic milestones. **a**–**b** Kidney biopsy N#3. **a** Massive tubulointerstitial infiltration (arrow) by small, morphologically unremarkable lymphocytes (hematoxylin and eosin stain, original magnification × 200). Distended tubulus is pointed by dashed arrow and preserved glomerulus by arrowhead. **b** Immunohistochemical stain showing predominance of CD8 positive T-cells (brown color, original magnification × 200). **c**–**e** Skin biopsy. **c** Dermal infiltration by small, morphologically unremarkable lymphocytes. **d** Immunohistochemical stain showing predominance of CD8 positive T-cells (brown color). **e** Proliferation fraction < 5% measured by staining for Ki-67 (brown color). **f** Image and distribution of skin rash. **g** PCR analyses of TCRB (Dβ–Jβ) gene rearrangements were performed using the BIOMED-2 multiplex PCR protocol, purchased from InVivoScribe Technologies, San Diego, CA. The prominent peaks of the same base pair size (green) demonstrate identical incomplete monoclonal rearrangement of *TCRB* in the kidney (upper), skin (middle), and blood (lower)

As his kidney function increasingly deteriorated, immunosuppressive treatment to control renal inflammation was started including corticosteroids and mycophenolate mofetil (Supplementary Table). Recurrent respiratory infections required repeated antimicrobial courses, physiotherapy, and an increase in the administered Ig dose (Supplementary Table). Transient improvement of kidney function was observed, but subsequent kidney biopsy (N#4) showed a progression of tubulointerstitial damage leading eventually to lymphoablative treatment with alemtuzumab (anti-CD52). Depletion of peripheral lymphocytes was achieved in the subsequent 6 months, but without any histopathologic improvement in the kidney biopsy (N#5).

Moreover, a few weeks following alemtuzumab therapy, the boy presented with increasing skin rash distributed over large areas (up to 25%) of the trunk (Fig. 1f). Investigation of the skin biopsy revealed a dermal infiltrate of small T-cells with a cytotoxic phenotype (Fig. 1c–d), identical to that previously found in the kidney and bone marrow. The proliferation fraction as measured by staining for Ki-67 was extremely low < 5% (Fig. 1e). Moreover, PCR analysis showed that the clone in the skin was the same as that in the kidney and bone marrow (Fig. 1g, middle panel). There is a reported case of clonal CD8-positive T-cell-rich lympho-histiocytic dermatitis in an adult patient with XLA responding to topical corticosteroids alone [2]. In our case, however, we did not observe improvement, but rather an increase of the skin infiltrates upon topical corticosteroid treatment.

Finally, Aichi virus (AiV) was detected by the metagenomic sequencing of the skin biopsy sample. A member of the *Picornaviridae* family, human AiV, replicates in the gastrointestinal tract, resulting usually in subclinical infections in immunocompetent individuals, with high seroprevalence in adults globally. High prevalence of viral excretion in stool samples from patients with human immunodeficiency virus indicates its opportunistic behavior in this population with underlying T-cell defects [3]. Chronic infection with AiV has been reported in one pediatric XLA patient with severe multi-organ disease that, similar to our case, included renal dysfunction [4]. That patient was offered HCT with an HLA-matched sibling donor to control the viral infection [5] and was cured from AiV. However, acute and chronic graft-versus-host disease (GvHD) was observed and required post-HCT immunosuppressive treatment. Our patient did not respond to different modes of immunosuppression, and in the absence of potent anti-viral agents against AiV, we considered HCT. We chose to perform a haplo-HCT with ex vivo T-cell receptor alpha beta (TcRαβ)-depleted cells from a parental donor for two reasons. First, haplo-HCT with a TcRαβ-depleted graft most often does not require any post-HCT pharmacological immunosuppression for GvHD prophylaxis. This was an important factor because our patient already had drastically affected kidney function and many of the immunosuppressive agents, such as cyclosporin A, are nephrotoxic. Second, due to the progressive deterioration in kidney function, there is a potential risk that the patient will require a kidney transplant. In such case, the successful kidney transplantation from the same parental haploidentical HCT donor would not require life-long immunosuppression.

Four years after the first signs of renal function deterioration, the patient started conditioning therapy with anti-thymocyte globulin Grafalon 45 mg/kg, fludarabine 160 mg/m^2^, treosulfan 42 mg/m^2^, and thiotepa 10 mg/kg. Due to suboptimal donor harvest, the patient was grafted with CMV-seropositive-matched maternal haploidentical cells on 2 consecutive days, with a total of 18.6 × 10^6^ CD34^+^ cells/kg. For EBV-reactivation prophylaxis, one dose of B-cell depleting rituximab 375 mg/m^2^ was administered 1 day post HCT. As TcRαβ-depletion was efficient, the patient did not receive further GvHD prophylaxis. The patient never experienced any severe complications from the conditioning treatment. On day + 12 after haplo-HCT, thrombocytes had recovered to > 50 × 10^9^/L; on day + 13, neutrophils had recovered to > 0.5 × 10^6^/L, and the patient was discharged from the hospital on day + 15 in good clinical condition. At that time point, and thereafter, the patient has displayed complete donor chimerism. Details on immune reconstitution are shown in the Supplementary Fig. 1.The patient’s life-long productive chronic cough and the T-cell skin infiltrates disappeared within 1 month after HCT. Lymphoid recovery is typically slow after haplo-HCT, but the patient experienced no infectious complications after HCT. Immunoglobulins were supplemented 6 times up to 6.5 months after HCT. The patient had SARS-CoV-2 antibodies following immunization despite low concentrations of CD19^+^ B-cells, at 0.02 × 10^9^/L; however as this sample was taken 2 months after the last Ig supplementation, we cannot fully exclude these antibodies to derive from the supplementation treatment. CD4^+^ lymphocyte concentrations exceeded 0.2 × 10^9^/L 9 months after HCT, and after 14 months, CD8^+^ cells remain low at 0.18 × 10^9^/L. One year after haplo-HCT, IgG and CD19 + levels were 0.86 g/L and 0.04 × 10^9^/L, respectively. Levels then increased spontaneously to IgG levels of 1.65 g/L at 15 months, and CD19 + concentration to 0.09 × 10^9^/L (normal range 0.07–0.46 × 10^9^/L), of which 90% were naïve B-cells. After 18 months, IgG (1.78 g/L) and IgA (0.14 g/L) levels remained low, however stable, and IgM levels had normalized to 0.49 g/L and we considered the patient also cured from the XLA. Kidney function was stable until 10 months after transplantation; whereafter, GFR measured by iohexol clearance decreased from 46 to 33 mL/min/1.7 m^2^ and has since been stable around 33–37 mL/min/1.7 m^2^. A new kidney biopsy (N#6) showed severely affected histology with global glomerulosclerosis, interstitial fibrosis, and tubulointerstitial inflammation. Flow cytometry and PCR analysis of lymphocyte infiltrates could not confirm residual T-cells of the original clone, and infiltrates were judged to be reactive tissue damage (Supplementary Fig. 2).

In conclusion, we describe the second pediatric patient with XLA who developed a chronic and disseminated AiV infection requiring HCT for virus control. Interestingly, both cases had AiV-induced renal dysfunction, and in both cases, lymphoma was considered in the differential diagnosis, based on the massive, monoclonal lymphocytic infiltration. Lack of a routinely available method of AiV screening did in fact delay the proper diagnosis in our patient, and these two cases suggest that chronic AiV infection in XLA patients may be more common than anticipated. Hence, extended diagnostic testing with fore-front molecular tools, such as metagenomic analysis, should be considered in this type of patients when presenting with multi-organ disease possibly caused by an underlying infection, and when a pathogen cannot be identified with standard routine investigations. Performing the first TcRαβ-depleted haplo-HCT proves the feasibility for subsequent viral control and suggests it is beneficial, due to the lack of any requirement for renal-toxic pharmacological immunosuppression as well as the fact that a haploidentical donor may be an attractive kidney donor when kidney transplantation is required. Lastly, our case benefitted from the allogeneic HCT since many of the XLA-associated symptoms, that were not related to the AiV, disappeared, and the patient became independent of Ig-supplementation. This illustrates a role for HCT at least in a selection of XLA patients.

## Supplementary Information

Below is the link to the electronic supplementary material.

Supplementary file1 (PDF 163 KB)

Supplementary file2 (PDF 964 KB)

Supplementary file3 (DOCX 18 KB)

## Data Availability

Not applicable.
