# Supplementary material for: Immunologic Control of Disseminated Aichi Virus Infection in X-Linked Agammaglobulinemia by Transplantation of TcRαβ-Depleted Haploidentical Hematopoietic Cells
Source: J Clin Immunol. 2022 Jul 5;42(7):1401–4. doi: 10.1007/s10875-022-01314-5 (PMC9253251; doi:10.1007/s10875-022-01314-5)
Supplement: Supplementary file 1 — Supplementary file1 (PDF 163 KB) [file 10875_2022_1314_MOESM1_ESM.pdf]

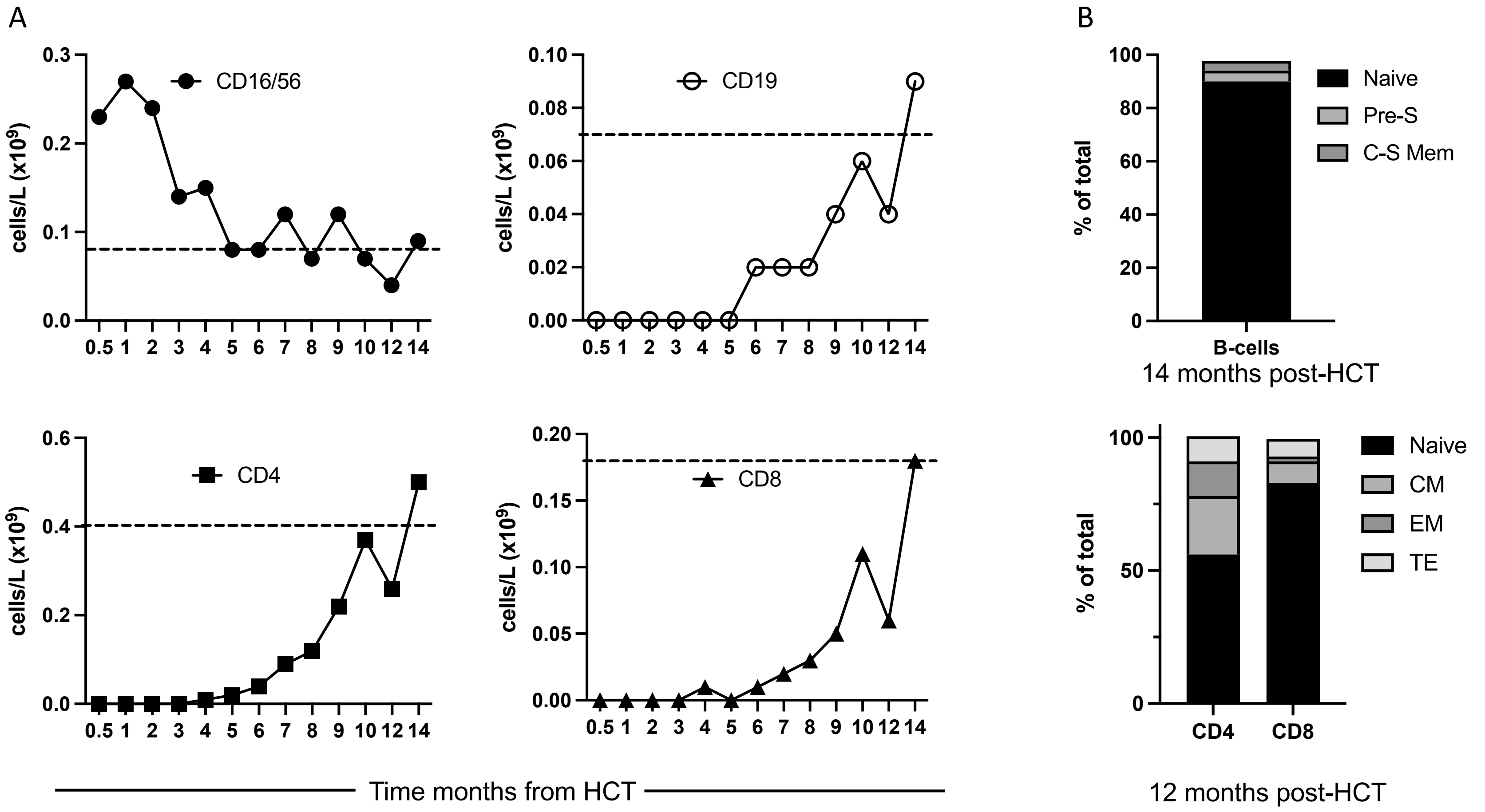

Supplementary Figure 1. Immune reconstitution after HCT. A) Absolute levels of CD16/56 NK-cells, CD19 B-cells, CD4 T-cells and CD8 T-cells at indicated time-points after HCT. The dashed horizontal lines indicates the lower normal limit. B. Composition of B- (upper) and T-cell(lower) subsets at 14 and 12 months after HCT, respectively. Pre-S Pre-switch; C-S Mem Class-switch memory; CM Central memory; EM Effector memory; TE Terminal effector
