# Supplementary material for: Immunologic Control of Disseminated Aichi Virus Infection in X-Linked Agammaglobulinemia by Transplantation of TcRαβ-Depleted Haploidentical Hematopoietic Cells
Source: J Clin Immunol. 2022 Jul 5;42(7):1401–4. doi: 10.1007/s10875-022-01314-5 (PMC9253251; doi:10.1007/s10875-022-01314-5)
Supplement: Supplementary file 2 — Supplementary file2 (PDF 964 KB) [file 10875_2022_1314_MOESM2_ESM.pdf]

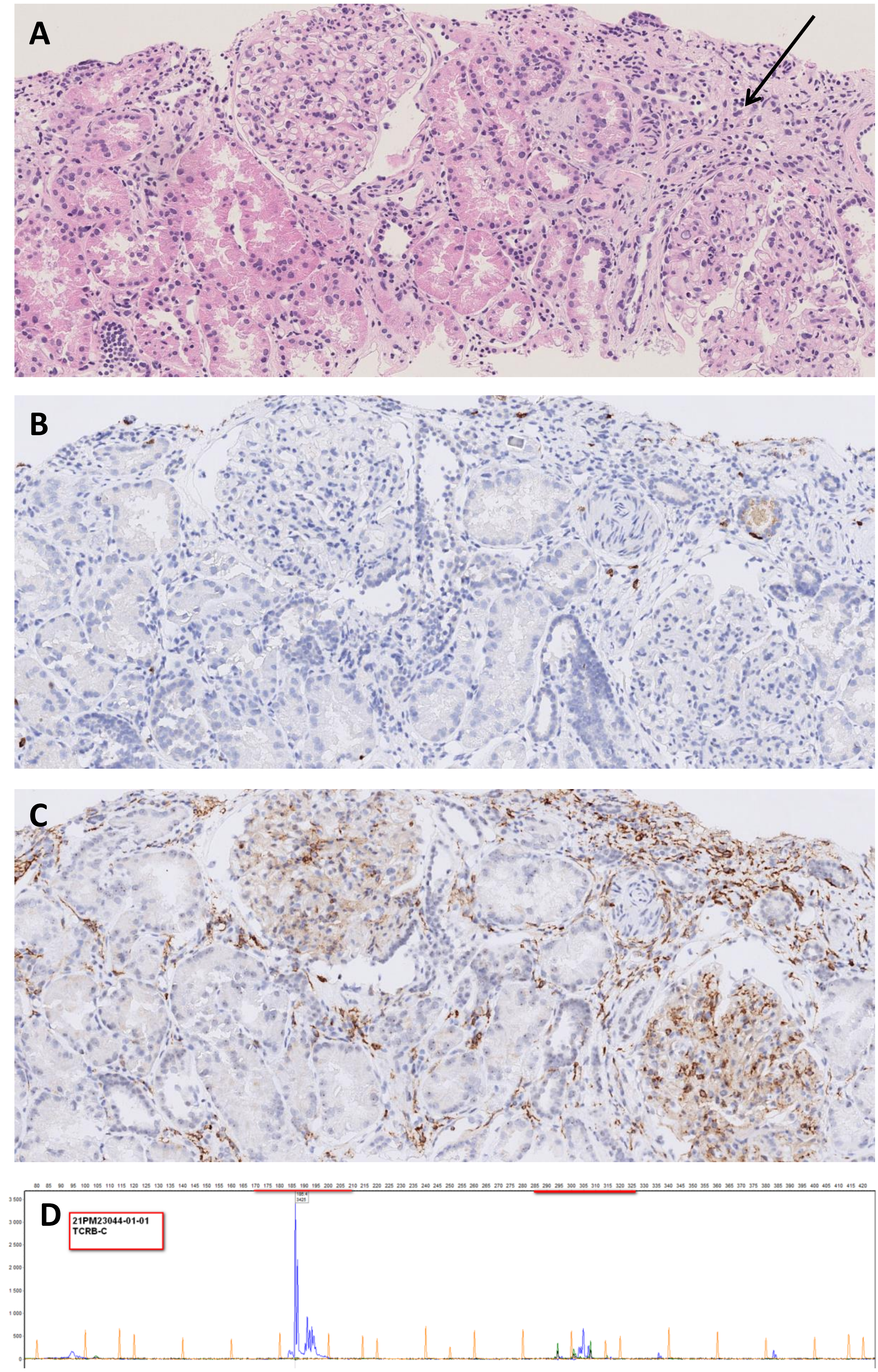

Supplementary Figure 2. Kidney biopsy post-HCT. **A**) Some residual tubolointerstitial inflammation with fibrosis (arrow). Hematoxylin and eosin stain, original magnification x200. **B**) Immunohistochemical stain showing very few CD8 positive T-cells (brown color, original magnification x200). **C**) Immunohistochemical stain showing CD4 positive T-cells composing inflammatory infiltrates (brown color, original magnification x200). **D**) PCR analysis of the kidney showing an incomplete monoclonal rearrangement of *TCRB* (smaller peaks), different from the pre-transplant analysis, and interpreted as reactive.
