# Supplementary material for: Immunologic Control of Disseminated Aichi Virus Infection in X-Linked Agammaglobulinemia by Transplantation of TcRαβ-Depleted Haploidentical Hematopoietic Cells
Source: J Clin Immunol. 2022 Jul 5;42(7):1401–4. doi: 10.1007/s10875-022-01314-5 (PMC9253251; doi:10.1007/s10875-022-01314-5)
Supplement: Supplementary file 3 — Supplementary file3 (DOCX 18 KB) [file 10875_2022_1314_MOESM3_ESM.docx]

**Supplementary table** Overview of diagnostic workup, GFR, IgG levels, IgG replacement and immunosuppressive treatment prior to, and after haplo-HCT (autumn 2020)

|  | 2016 | 2017 | 2018 | 2019 | 2020 | 2021 |
| --- | --- | --- | --- | --- | --- | --- |
| Diagnostic workup for pathogens  (all negative) | Bacterial culture of the urine  HBsAg  HCV-Ab  HIV-Ag, and Ab in the blood | Fungal culture of the urine  PCR-DNA for CMV, EBV, adenovirus, BK-virus, JC-virus in urine  PCR-DNA for EBV, HHV-8, PCR-RNA and Ab for puumalavirus in the blood  Enterovirus in feces | Immunostaining for CMV and BK-virus in the kidney biopsy  TB-specific Ag, PCR-RNA for HIV-1, HCV and HBV in the blood |  | Metagenomic sequencing in the serum |  |
| GFR by iohexol clearence  Normal 86 -124  ml/min/1.7 m^2^ | 40 | 37 - 56 | 52 | 46 - 49 | 47 | 33 |
| IgG levels (mean)  Normal 6.1-14.5 g/L | 4.4  (3.6-6.0) | 8.2  (6.6-10.2) | 8.2  (3.9-13.6) | 7.3  (6.9-7.8) | 6.9  (4.9-12.0) | Described in the letter |
| IgG replacement  mg/kg bodyweight/week | 140 | 125 | 125-160 | 160-200 | 200 |  |
| Immuno-  suppressive  treatment |  | Methylprednisolone  15mg/kg x 3  Prednisolone  1.2 mg/kg x 1 for 4 weeks  Prednisolone  1.2 mg/kg x1 alternate day dosage for 6 months | Prednisolone for 12 months tapering from 1.2 mg/kg to 0.1 mg/kg alternate day dosage, stopped in december  MMF 600 mg/m^2^ x 2  AUC 82-196 mg/L/h  (30-60) for 7 months  Alemtuzumab total dose 60 mg, 1,25 mg/kg (12 mg given in five subsequent days) in december |  |  |  |

GFR: glomerular filtration rate; IgG: immunoglobulin G; HBsAg: hepatitis B virus surface antigen; HCV-ab hepatitis C virus antibodies; HIV-Ag and Ab: human immunodeficienty virus antigen and antibodies; PCR: Polymerase Chain Reaction; CMV: cytomegalovirus; EBV: Epstein-Barr-virus; HHV-8: human herpes virus-8; TB: tuberculosis; MMF: mycophenolate mofetil; AUC: area under curve
